# Supplementary material for: The Non-Cancer Specific Elevation of the Serum Squamous Cell Carcinoma Antigen during the Post-Radiotherapy Follow-Up of Cervical Cancer Patients
Source: Diagnostics (Basel). 2021 Aug 31;11(9):1585. doi: 10.3390/diagnostics11091585 (PMC8464782; doi:10.3390/diagnostics11091585)
Supplement: Supplementary file 1 [file diagnostics-11-01585-s001.zip › diagnostics-1313184-Supplementary Figures S1-S2.pdf]

Figure S1

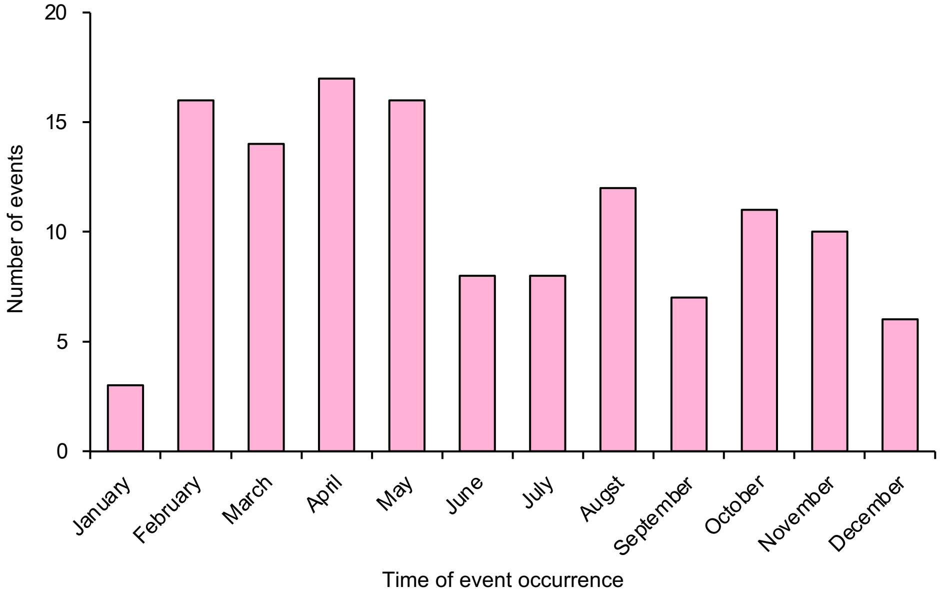

Figure S1. Seasonal time of occurrence of post-treatment non-cancer specific elevation of serum squamous cell carcinoma antigen in patients with cervical cancer patients treated with definitive radiotherapy (*n* = 128).

Figure S2

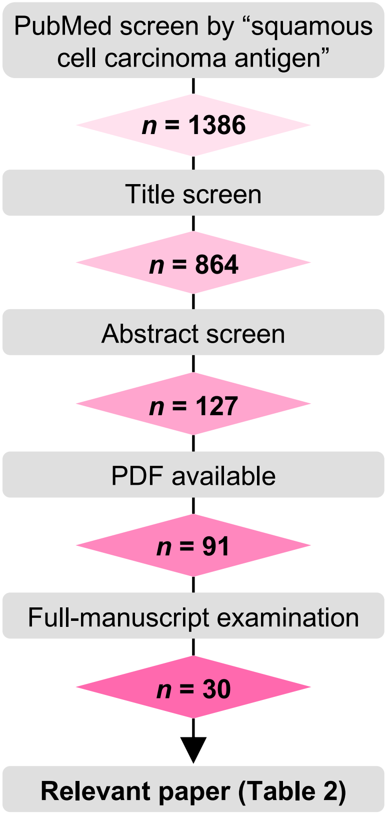

Figure S2. Flow diagram showing the systemic literature review of clinical factors potentially associated with non-cancer specific elevation of serum squamous cell carcinoma antigen levels.
