# Supplementary figures and images for: The Non-Cancer Specific Elevation of the Serum Squamous Cell Carcinoma Antigen during the Post-Radiotherapy Follow-Up of Cervical Cancer Patients
Source: Diagnostics (Basel). 2021 Aug 31;11(9):1585. doi: 10.3390/diagnostics11091585 (PMC8464782; doi:10.3390/diagnostics11091585)

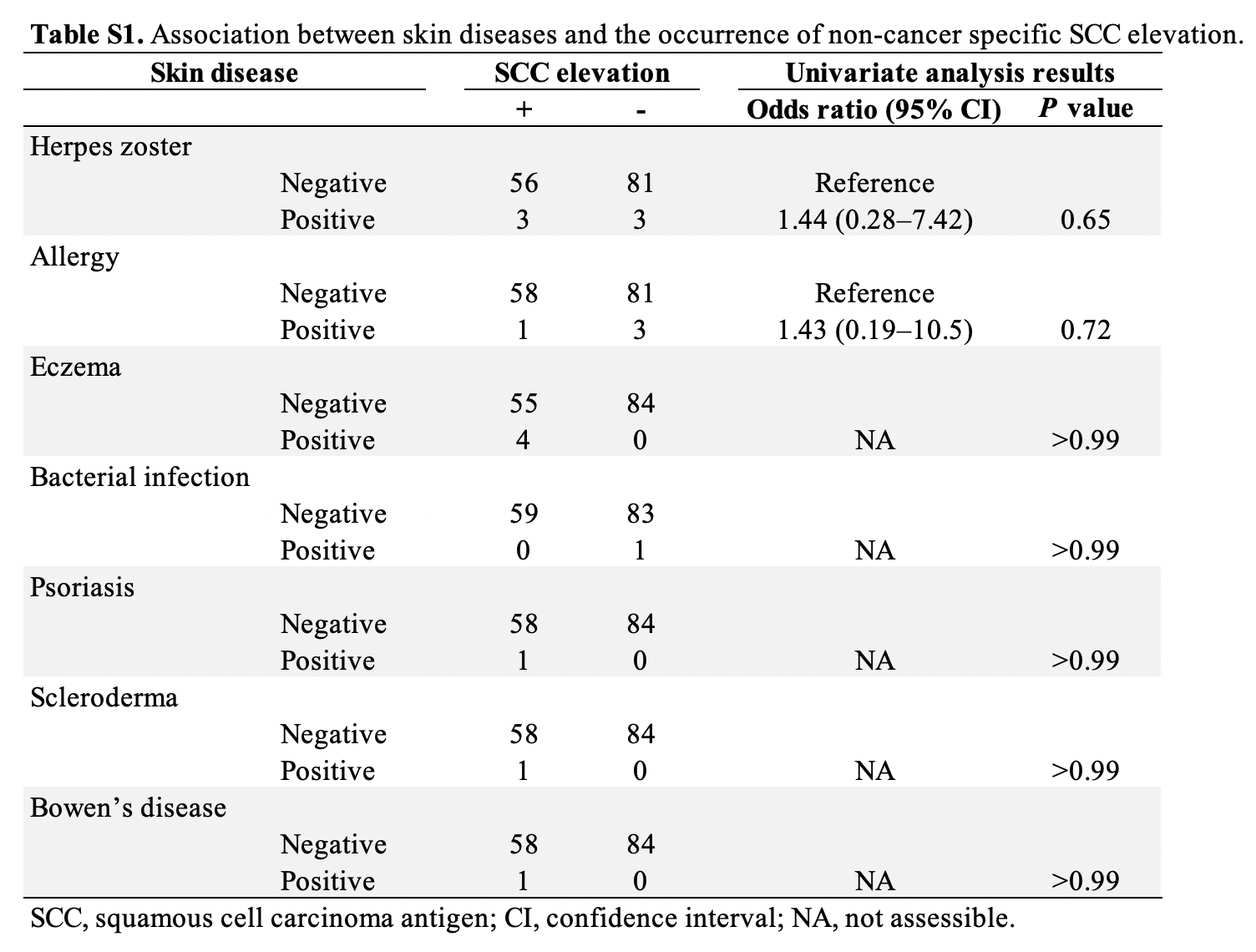

Supplement: Supplementary file 1 [file diagnostics-11-01585-s001.zip › diagnostics-1313184--Supplementary Table S1.png]
